# Sociodemographic factors associated with inadequate food group consumption and dietary diversity among infants and young children in Nepal

**DOI:** 10.1371/journal.pone.0213610

**Published:** 2019-03-11

**Authors:** Yeji Baek, Stanley Chitekwe

**Affiliations:** United Nations Children’s Fund (UNICEF) Nepal, UN House, Pulchowk, Kathmandu, Nepal; National Institute of Health, ITALY

## Abstract

Infants and young children need diversified diets to grow healthy. However, there is limited evidence on factors associated with consumption of various food groups. This study aimed to identify the sociodemographic factors associated with inadequate food group consumption and not meeting the minimum dietary diversity (MDD) among infants and young children aged 6–23 months in Nepal. Using cross-sectional data from the 2016 Nepal Demographic and Health Survey, the factors at the individual-, household-, and community-levels associated with not consuming foods from the seven food groups, which are grains, roots and tubers, legumes and nuts, dairy products, flesh foods, eggs, vitamin-A rich fruits and vegetables, and other fruits and vegetables, and not meeting the MDD were examined. The least consumed food group was eggs, followed by flesh foods and 46.5% of children received the MDD. Children aged 6–11 months had higher odds of not consuming foods from the seven food groups and not meeting the MDD than older children. Children from the poorest quintile had higher odds of not consuming legumes and nuts, dairy products, flesh foods, and other fruits and vegetables, and not meeting the MDD. Children from Terai/Madhesi Other had higher odds of not consuming foods from the seven food groups compared to those from the other groups. Children from Province 2 had higher odds of not consuming eggs, vitamin-A rich fruits and vegetables, and other fruits and vegetables, and not meeting the MDD. Dietary diversity among children in Nepal needs improvement. National policies and programs need to promote the consumption of diverse food groups by considering different sociodemographic characteristics.

## Introduction

Infant and young child feeding (IYCF) is critical for child health, development and survival [1, 2]. As infants reach 6 months of age, they need to begin consuming soft, semi-solid, and solid foods in addition to breast milk [3]. Complementary feeding is defined as the process starting when breast milk alone is no longer sufficient to meet the nutritional requirements of infants, therefore other foods and liquids are needed in addition to breast milk [1]. A lack of healthy complementary feeding practices is the main cause of undernutrition which is a direct cause of mortality [1]. Food must come from a variety of food groups to ensure that children receive all the vitamins, minerals, and nutrients they need to grow, develop, stay healthy, and reach their full potential [3]. The World Health Organization (WHO) recommends that complementary foods should be varied and include quantities of meat, eggs, vitamin A-rich fruits, and vegetables every day [4]. The Minimum Dietary Diversity (MDD) is one of the core indicators developed by the WHO to measure IYCF practices together with early initiation of breastfeeding; exclusive breastfeeding under 6 months; continued breastfeeding at 1 year; introduction of soft, semi-solid or solid foods; minimum meal frequency; minimum acceptable diet; and consumption of iron-rich or iron-fortified foods [2]. The MDD is defined as the proportion of children who receive foods from four or more food groups out of the following seven food groups, grains, roots and tubers, legumes and nuts, dairy products, flesh foods, eggs, vitamin-A rich fruits and vegetables, and other fruits and vegetables [2]. Dietary data from children 6–23 months of age in ten developing countries showed that consumption of foods from at least four food groups on the previous day would mean that the child had a high likelihood of consuming at least one animal-source food and at least one fruit or vegetable, in addition to a staple food [5].

A study analyzing the Demographic and Health Surveys (DHS) data from eleven countries concluded that dietary diversity is generally associated with child nutritional status and the association is independent of socioeconomic factors [6]. However, data showed that many infants and young children do not receive optimal feeding. Globally, only 29% of infants and young children 6–23 months of age met the criteria of dietary diversity [3]. There are a number of studies examining the factors associated with dietary diversity. Studies from Bangladesh [7], Indonesia [8], Sri Lanka [9], Pakistan [10], Ghana [11], and Tanzania [12] indicated that younger children and children from the poor household were less likely to meet the MDD. The association between poor maternal education and inadequate dietary diversity was documented in studies from Bangladesh [7], Indonesia [8], Sri Lanka [9], India [13, 14], and Ethiopia [15]. Similarly, a few studies were conducted to examine IYCF practices and identify the determinant factors in Nepal [16, 17, 18, 19,20]. Among studies that focused on dietary diversity [17, 18, 20], they found that children whose mothers had secondary or higher education were more likely to meet the MDD. Four or more antenatal clinic visits of mothers [17, 20], the top wealth quintile [17], mothers’ frequent exposure to media [17], the ages of mothers (≥35 years) [18], and the older ages of children (18–23 months) [20] were found to be associated with meeting the MDD. However, there is limited data on the consumption of each food group which directly determines dietary diversity. The proportion of each food group consumption and its trend were examined in the previous studies [17, 18, 20], but factors associated with the consumption of each food group are yet to be found. Rather than only focusing on the MDD, identifying the sociodemographic factors associated with the consumption of each food group would provide detailed information to help understand the current situation and identify underserved groups. In addition, by focusing on food groups individually, policy makers and health-care professionals would be able to target each food group to formulate adequate strategies and design intervention programs to improve dietary diversity and the nutritional status of infants and young children. Using the 2016 Nepal DHS, the latest national study, our study aims to identify the factors at the individual-, household-, and community-levels associated with inadequate food group consumption and dietary diversity among children aged 6–23 months in Nepal.

## Materials and methods

### Data source

We used data from the 2016 Nepal DHS for this study [21]. The DHS is a nationally representative household survey that provides comprehensive data about population, maternal and child health issues in Nepal. Data were collected from June 2016 to January 2017 on demographic indicators and maternal, child and family health, including maternal and child mortality, maternal and newborn care, nutritional status of women and children and reproductive health. Sampling was based on a multi-stage and stratified cluster. Detailed information on sample design is described in the 2016 Nepal DHS report [21]. All women aged 15–49 years from the selected households were eligible to be interviewed. A weighted total of 12,862 women were interviewed using a questionnaire, with a response rate of 98.3%. Of the 4,887 children under age 5 in the selected households, a weighted total of 1,497 children aged 6–23 months living with the respondents were included in the analysis to assess food group consumption and the MDD as data on complementary feeding were collected from children aged 6–23 months.

#### Ethics statement

Ethical approval for this study was not sought as this study was based on secondary data from the Nepal DHS 2016 with all identifying information removed. We obtained permission from The DHS Program for the use of the datasets.

### Study variables

#### Food group consumption and the minimum dietary diversity

The standardized questionnaire was used to assess food group consumption based on the DHS Program’s standard Demographic and Health Survey (DHS-7) and the WHO’s guideline [2, 21]. Mothers were asked whether the children ate any amount of food from seven food groups, including grains, roots and tubers, legumes and nuts, dairy products (milk, yogurt, and cheese), flesh foods (meat, fish, poultry and liver/organ meats), eggs, vitamin-A rich fruits and vegetables, and other fruits and vegetables the day before the interview. Each food group was described in the questionnaire with examples of food items. Mothers responded ‘Yes’ if the children ate any amount of food from each food group according to the WHO’s guideline regardless of its quantities or frequencies [2].

The MDD was estimated according to the WHO’s definition [2]. It is defined as the proportion of children 6–23 months of age who received foods from four or more out of seven food groups [2]. Detailed information on the questionnaire is described in the 2016 Nepal DHS [21].

#### Sociodemographic characteristics

The individual-level factors included age, gender, mother’s age, mother’s education, mother’s antenatal care visits, and mother’s frequency of listening to radio. At the household-level, the wealth index quintile was used in the study. The wealth index was constructed based on the number and kinds of consumer goods households own and housing characteristics such as source of drinking water, toilet facilities, and flooring materials [21]. The community-level factors included residence, ecological region, province, and caste/ethnic groups. Nepal is a country of diverse geographical and topographical regions consisting of mountains, hills and plains (Terai). The nation is divided into the following seven provinces, generally numbered from east to west: Province 1 covering the eastern area of Nepal, Province 2 in the southeastern areas, Province 3 and 4 along the central belt, Province 5 covering the southwestern and hilly regions and Provinces 6 and 7 across the mid-far western regions. Caste/ethnic groups were categorized into Brahman/Chhetri, Terai/Madhesi Other, Dalits, Newar, Janajati, and Muslim [22]. Nepal consists of more than one hundred caste/ethnic groups. Although caste-based discrimination was officially abolished, social hierarchies based on ethnicity and caste are still reported as distinct features of Nepalese society [23, 24, 25]. It was reported that Madhesi, Dalits, and Janajati remain on the margins or stay at the bottom of the hierarchy, whereas Brahman/Chhetri are at the top [25].

### Statistical analysis

All statistical analyses were performed using the SAS University Edition. ‘Proc survey’ procedures were used to account for the complex survey design of the DHS, including stratification, cluster design, and weighting. Dependent variables were consumption of grains, roots and tubers, legumes and nuts, dairy products, flesh foods, eggs, vitamin-A rich fruits and vegetables, and other fruits and vegetables and the MDD. Independent variables, including age, gender, mother’s age, mother’s education, mother’s antenatal care visits, mother’s frequency of listening to radio, the wealth index quintile, residence, ecological region, province, and caste/ethnic groups were selected based on findings of previous studies [7–20] and their availability in the DHS dataset.

Descriptive statistics were performed to estimate the proportion of consumption of each food group and the MDD. To study the association between dependent variables and sociodemographic factors, bivariate and multiple logistic regressions were performed. Independent variables that were significantly associated with the dependent variables at p-value < 0.20 in bivariate logistic regression models were included in the multiple logistic regression models to estimate adjusted odds ratios for each dependent variable. Groups with highest consumption were selected as the reference groups.

Data were presented as means and standard errors for descriptive statistics and as odds ratios and 95% confidence intervals (CI) for logistic regressions. The statistical significance level was set as p-value < 0.05.

## Results

### Characteristics of the sample

The sociodemographic characteristics of a weighted total of 1,497 children (boys = 809; girls = 688) are described in Table 1. 33.7% of the children were aged 12–17 months and more than half were boys (54.1%). 29.2% of the children were from Janajati, 25.4% were from Brahmin/Chhetri, 20.4% were from Terai/Madhesi Other, 14.1 were from Dalits, 7.3% were from Muslim and 3.7% were of Newari origin.

**Table 1:** Sociodemographic characteristics of Nepalese children aged 6 to 23 months.

		N	%
Individual-level factors			
Age	6–11 months	499	33.3
	12–17 months	504	33.7
	18–23 months	494	33.0
Gender	Boy	809	54.1
	Girl	688	45.9
Mother’s age	15–24 years	774	51.7
	25–34 years	638	42.6
	35–49 years	84	5.6
Mother’s education	None	452	30.2
	Primary	296	19.8
	Secondary	405	27.1
	Higher	344	23.0
Mother’s antenatal care visits	None	58	3.9
	1–3	382	25.6
	4≥	1055	70.6
Mother’s frequency of listening to radio	None or less than once a week	1135	75.8
	At least once a week	362	24.2
Household-level factors			
Wealth quintile	Poorest	303	20.2
	Second	327	21.9
	Middle	341	22.8
	Fourth	318	21.2
	Richest	208	13.9
Community-level factors			
Residence	Urban	804	53.7
	Rural	693	46.3
Ecological region	Mountain	98	6.6
	Hill	576	38.5
	Terai	822	54.9
Province	Province 1	269	18.0
	Province 2	381	25.5
	Province 3	241	16.1
	Province 4	125	8.4
	Province 5	275	18.4
	Province 6	86	5.8
	Province 7	120	8.0
Caste/ethnic groups	Brahmin/Chhetri	379	25.4
	Terai/Madhesi Other	304	20.4
	Dalits	210	14.1
	Newar	55	3.7
	Janajati	436	29.2
	Muslim	109	7.3

Weighted

### Food group consumption and the minimum dietary diversity

**Fig 1:**
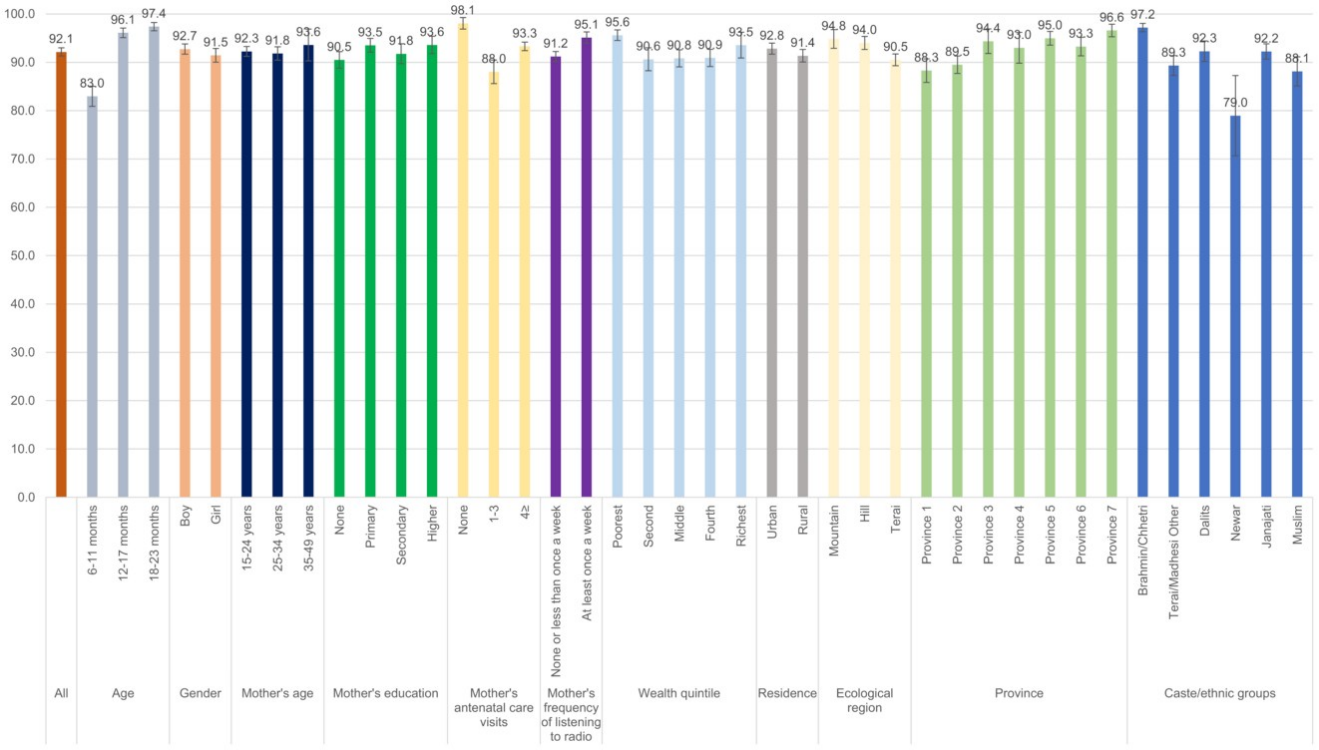
Percentage of Nepalese children aged 6 to 23 months consuming grains, roots and tubers by sociodemographic factors.

**Fig 2:**
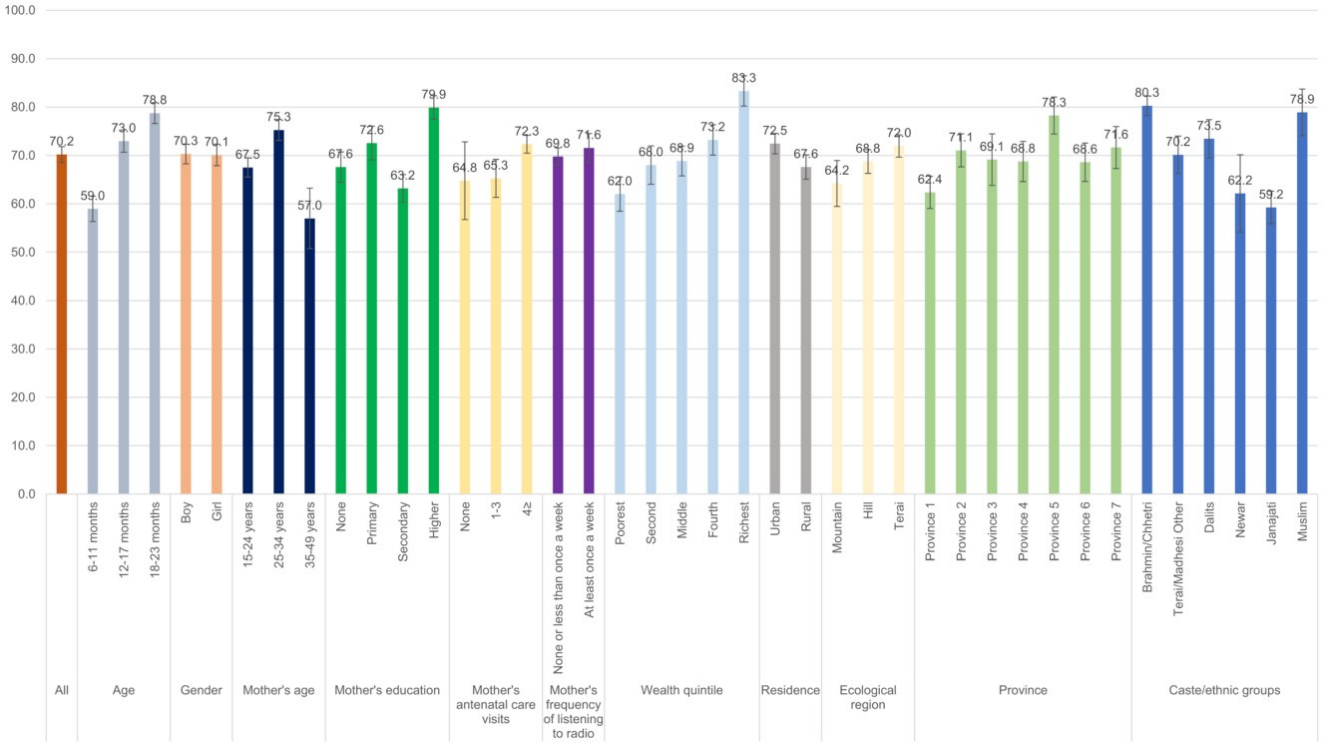
Percentage of Nepalese children aged 6 to 23 months consuming legumes and nuts by sociodemographic factors.

**Fig 3:**
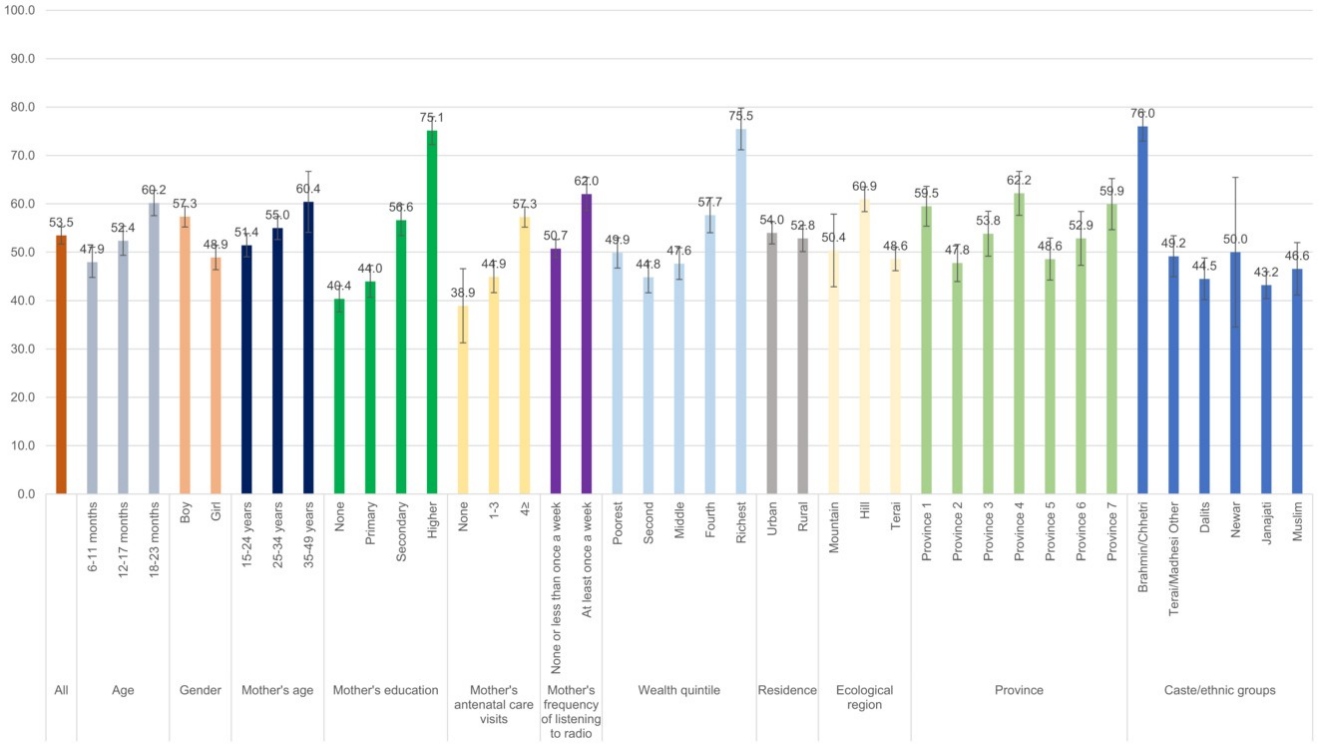
Percentage of Nepalese children aged 6 to 23 months consuming dairy products by sociodemographic factors.

**Fig 4:**
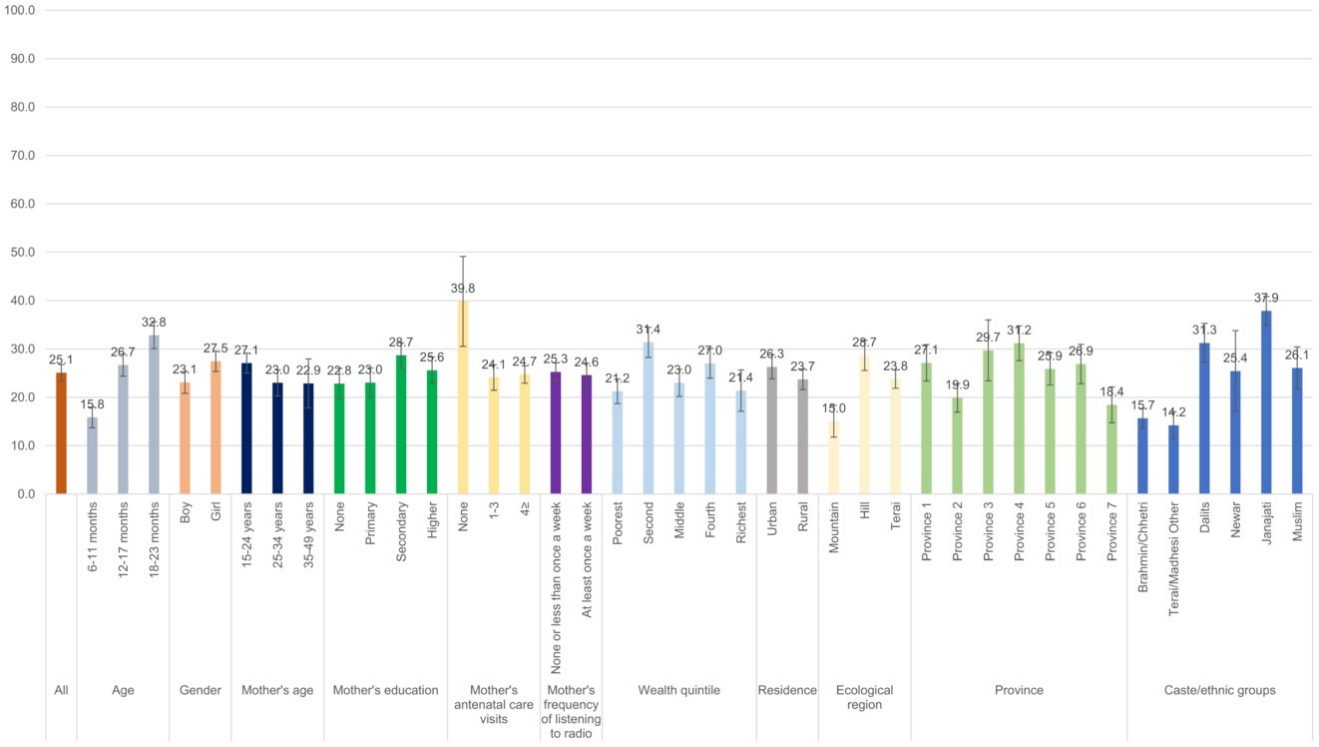
Percentage of Nepalese children aged 6 to 23 months consuming flesh foods by sociodemographic factors.

**Fig 5:**
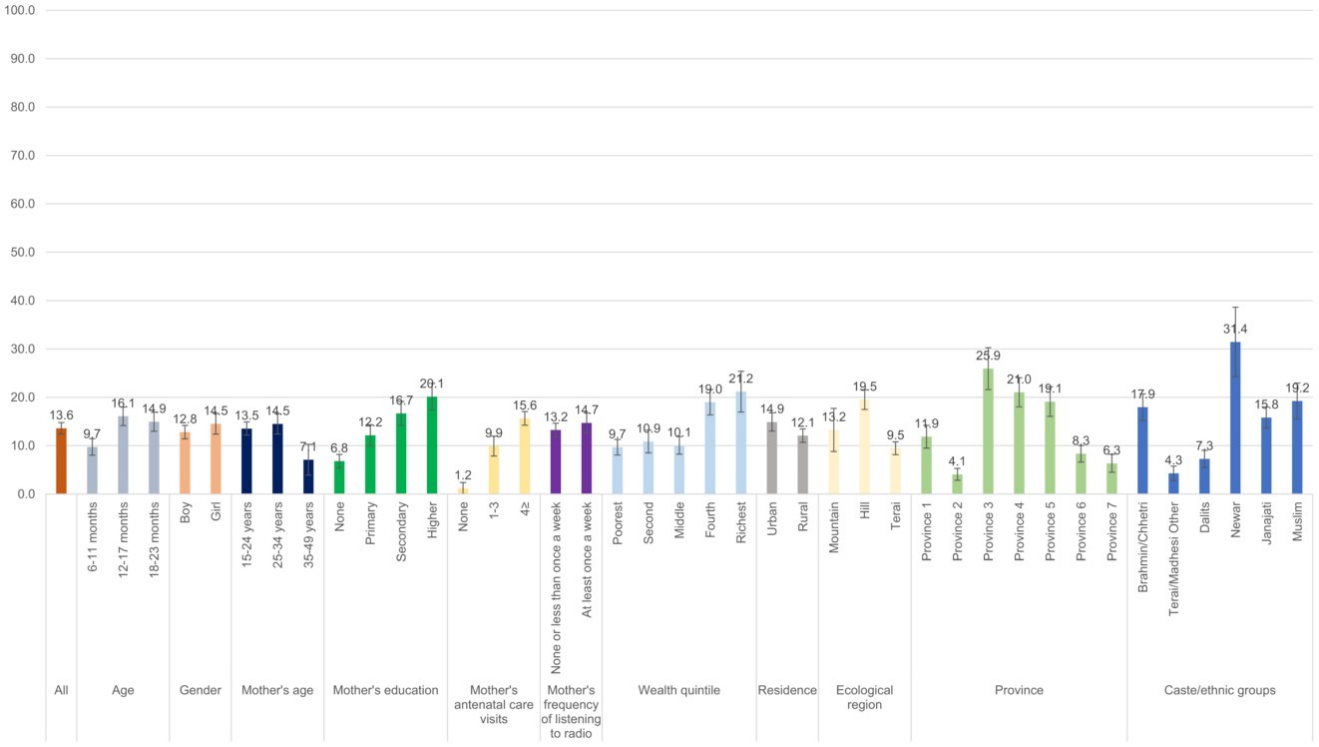
Percentage of Nepalese children aged 6 to 23 months consuming eggs by sociodemographic factors.

**Fig 6:**
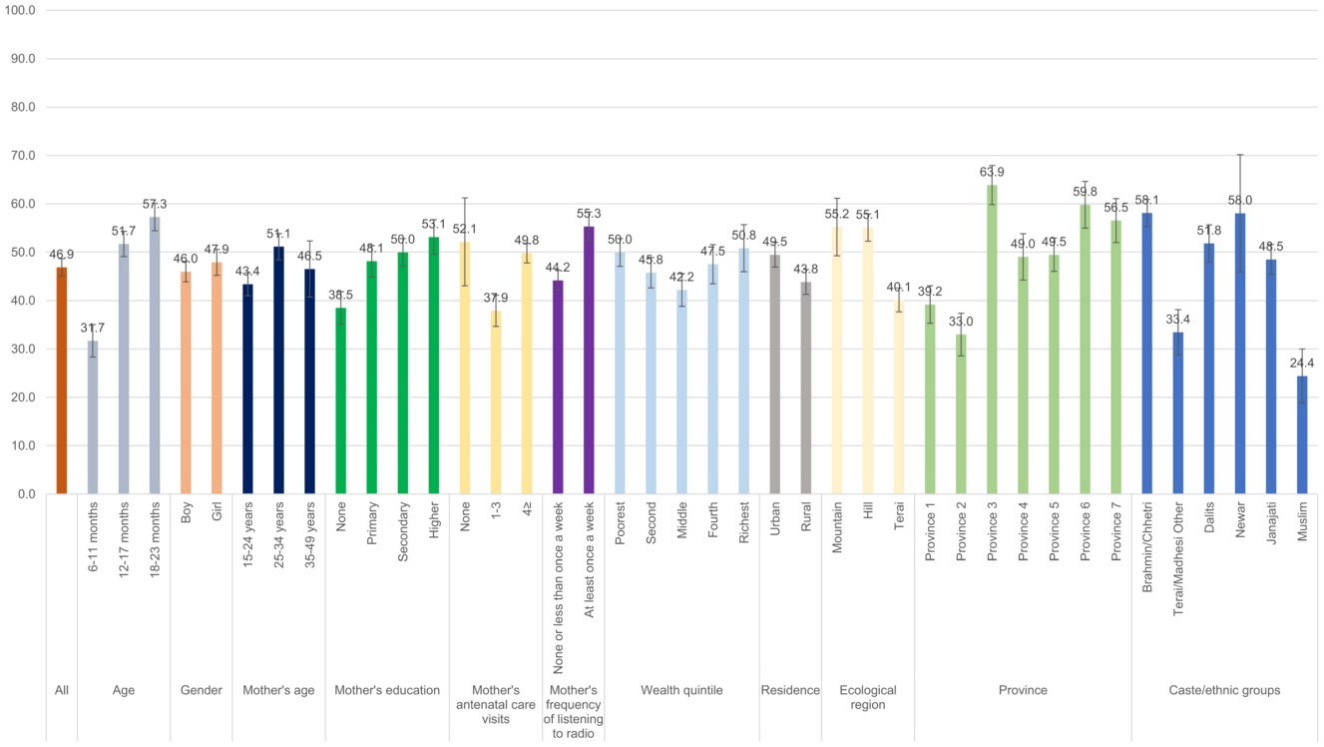
Percentage of Nepalese children aged 6 to 23 months consuming vitamin-A rich fruits and vegetables by sociodemographic factors.

**Fig 7:**
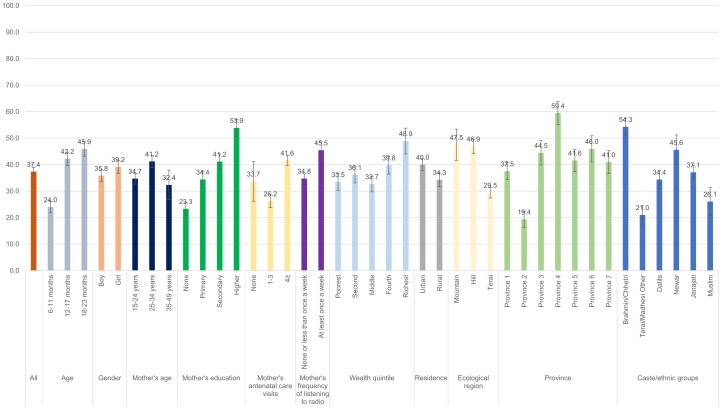
Percentage of Nepalese children aged 6 to 23 months consuming other fruits and vegetables by sociodemographic factors.

**Fig 8:**
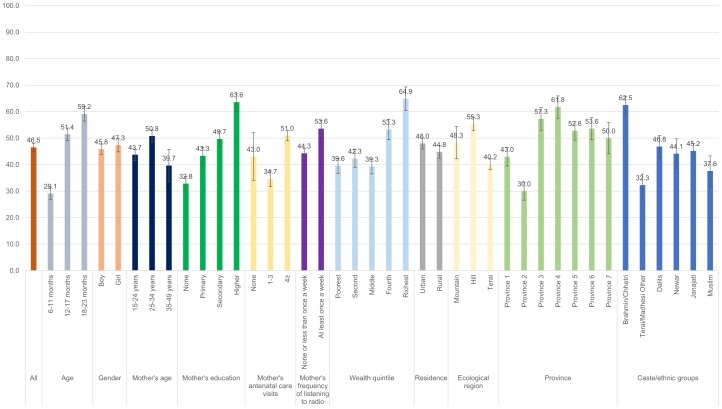
Percentage of Nepalese children aged 6 to 23 months meeting the minimum dietary diversity by sociodemographic factors.

Figs 1–8 show the average percentage of children consuming each seven food groups and meeting the dietary diversity by sociodemographic factors. The average percentage of consumption of grains, roots, and tubers was 92.1% (Fig 1). 70.2% of children consumed legumes and nuts (Fig 2) and 53.5% of children consumed dairy products (Fig 3). The average percentage of children who consumed flesh foods and eggs was only 25.1% (Fig 4) and 13.6% (Fig 5), respectively. Eggs were the least consumed food group. 46.9% of children consumed vitamin-A rich fruits and vegetables (Fig 6) and 37.4% of children consumed other fruits and vegetables (Fig 7). Overall, the average percentages of consumption amongst different food groups were lower among children 6–11 months than the 12–17 months or 18–23 months age groups. The consumption of different food groups was higher among children whose mothers had secondary and higher education. Except for flesh foods, children from Brahmin/Chhetri more likely received the other food groups. Less than half (46.5%) of the children received the MDD (Fig 8).

### Sociodemographic factors associated with inadequate food group consumption and dietary diversity

#### Grains, roots and tubers

**Fig 9:**
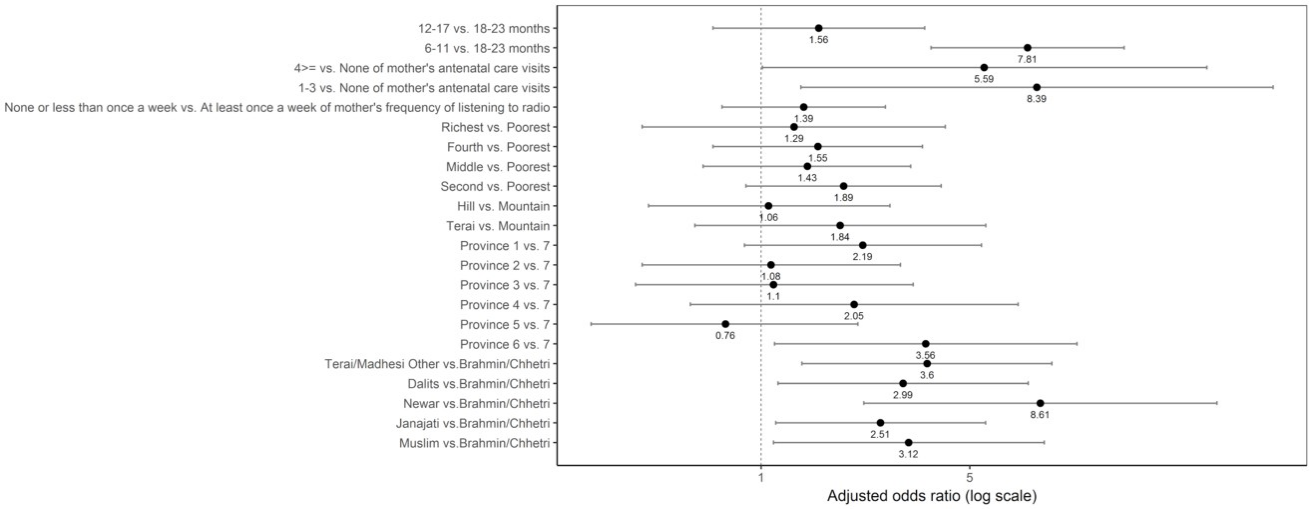
Odds ratio plot with 95% confidence intervals for not consuming grains, roots and tubers among Nepalese children aged 6 to 23 months.

Fig 9 shows that age, mother’s antenatal care visits, province, and caste/ethnic groups were associated with not consuming grains, roots and tubers after adjusting for the other variables. The odds of not consuming grains, roots and tubers was higher among children aged 6–11 months as compared to those aged 18–23 months, with an adjusted odds ratio of 7.81 (95% CI 3.71, 16.44). Children from Province 6 had higher odds of not consuming grains, roots and tubers than those from Province 7 (adjusted OR = 3.56; 95% CI 1.11, 11.42). Compared to children from Brahmin/Chhetri, those from the other caste/ethnic groups had higher odds of not consuming grains, roots and tubers (adjusted OR for Janajati = 2.51; 95% CI 1.12, 5.65, adjusted OR for Dalits = 2.99; 95% CI 1.14, 7.84, adjusted OR for Muslim = 3.12; 95% CI 1.10, 8.88, adjusted OR for Terai/Madhesi Other = 3.60; 95% CI 1.37, 9.42, and adjusted OR for Newar = 8.61; 95% CI 2.21, 33.60).

#### Legumes and nuts

**Fig 10:**
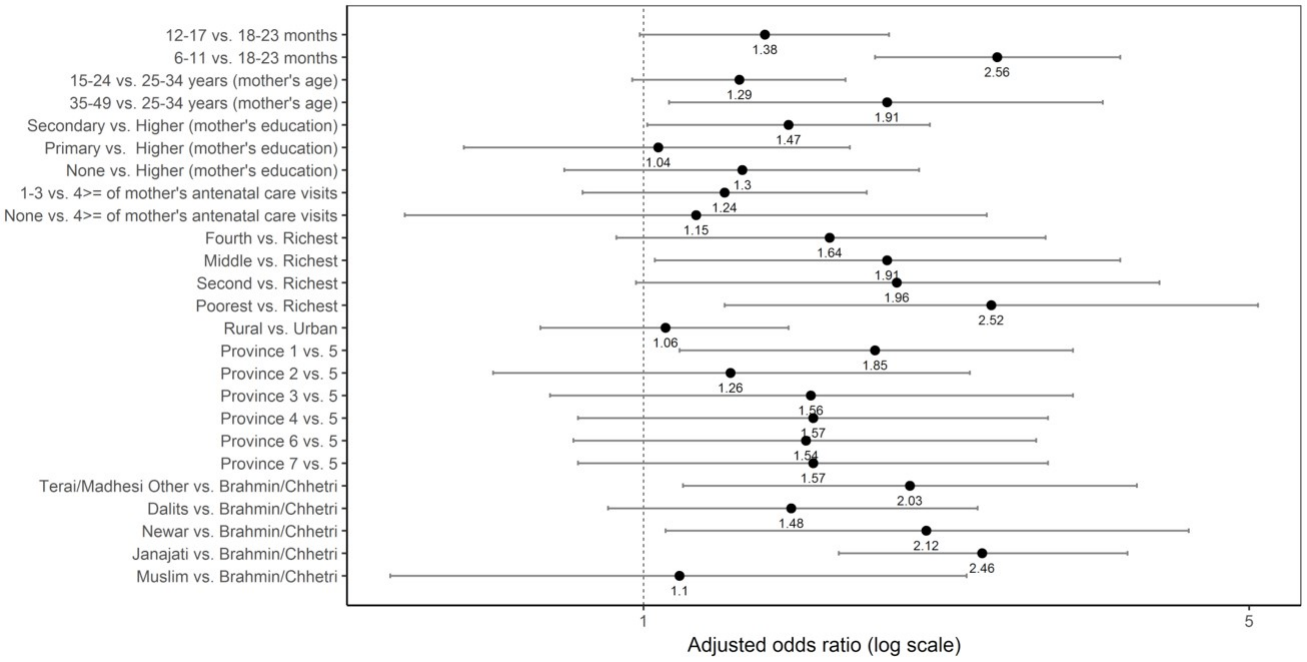
Odds ratio plot with 95% confidence intervals for not consuming legumes and nuts among Nepalese children aged 6 to 23 months.

Age, mother’s age, mother’s education, the wealth quintile, province, and caste/ethnic groups were significantly associated with not consuming legumes and nuts after adjusting for the other variables (Fig 10). Children 6–11 months old had higher odds of not consuming legumes and nuts compared to those 18–23 months old (adjusted OR = 2.56; 95% CI 1.85, 3.55). The odds of not consuming legumes and nuts was higher among children in the middle quintile (adjusted OR = 1.91; 95% CI 1.03, 3.55) and in the poorest quintile (adjusted OR = 2.52; 95% CI 1.24, 5.12) than those in the richest quintile. Compared with children from Province 5, the odds of not consuming legumes and nuts was higher among children from Province 1 (adjusted OR = 1.85; 95% CI 1.10, 3.13). The odds of not consuming legumes and nuts was higher among children from Terai/Madhesi Other (adjusted OR = 2.03; 95% CI 1.11, 3.71), Newar (adjusted OR = 2.12; 95% CI 1.06, 4.26), and Janajati (adjusted OR = 2.46; 95% CI 1.68, 3.62) than those from Brahmin/Chhetri.

#### Dairy products

**Fig 11:**
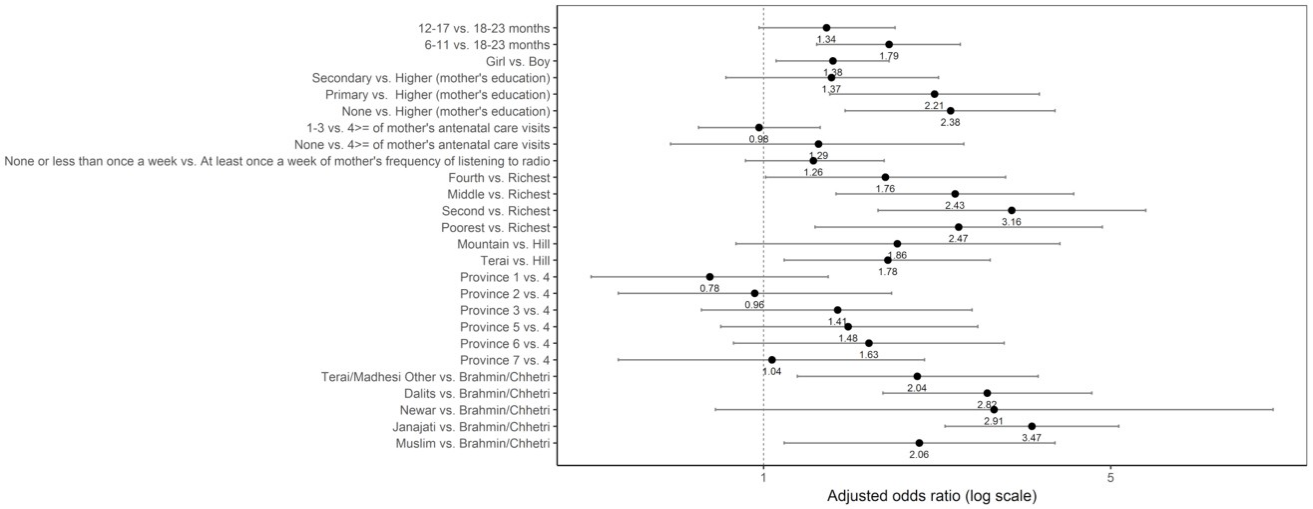
Odds ratio plot with 95% confidence intervals for not consuming dairy products among Nepalese children aged 6 to 23 months.

Sociodemographic characteristics significantly associated with not consuming dairy products were age, gender, mother’s education, the wealth quintile, ecological region, and caste/ethnic groups after adjusting for the other variables (Fig 11). The odds of not consuming dairy products was higher among children aged 6–11 months as compared to those aged 18–23 months (adjusted OR = 1.79; 95% CI 1.28, 2.49). Children in the fourth quintile (adjusted OR = 1.76; 95% CI 1.01, 3.07), middle quintile (adjusted OR = 2.43; 95% CI 1.40, 4.21), second quintile (adjusted OR = 3.16; 95% CI 1.70, 5.88), and poorest quintile (adjusted OR = 2.47; 95% CI 1.27, 4.81) had higher odds of not consuming dairy products compared to those in the richest quintile. The odds of not consuming dairy products was higher among children from Terai/Madhesi Other (adjusted OR = 2.04; 95% CI 1.17, 3.57), Dalits (adjusted OR = 2.82; 95% CI 1.74, 4.58), Janajati (adjusted OR = 3.47; 95% CI 2.32, 5.19), and Muslim (adjusted OR = 2.06; 95% CI 1.10, 3.86) than those from Brahmin/Chhetri.

#### Flesh foods

**Fig 12:**
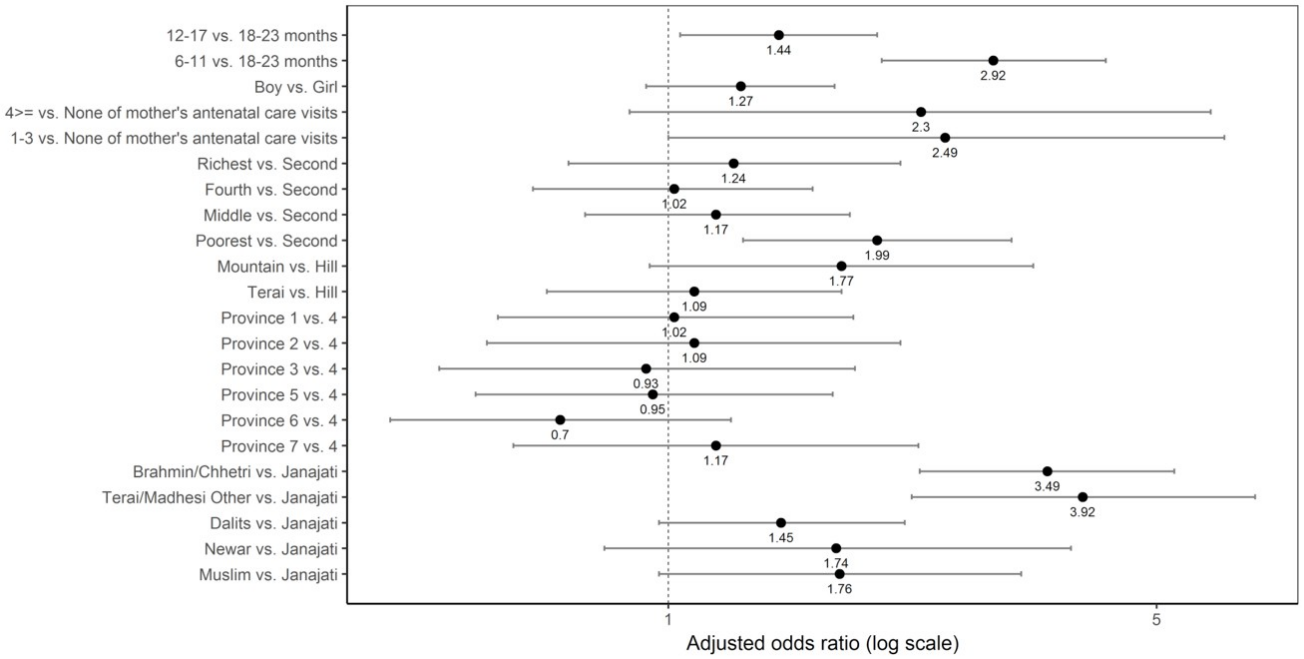
Odds ratio plot with 95% confidence intervals for not consuming flesh foods among Nepalese children aged 6 to 23 months.

Age, the wealth quintile, and caste/ethnic groups were significantly associated with not consuming flesh foods after adjusting for the other variables (Fig 12). Children 6–11 months old had higher odds of not consuming flesh foods compared to those 18–23 months old (adjusted OR = 2.92; 95% CI 2.02–4.23). The odds of not consuming flesh foods was significantly higher among children in the poorest quintile compared to those in the richest quintile (adjusted OR = 1.99; 95% CI 1.28–3.10). Compared to children from Janajati, those from Brahmin/Chhetri and Terai/Madhesi Other had higher odds of not consuming flesh foods, with an adjusted odds ratio of 3.49 (95% CI: 2.29, 5.30) and 3.92 (95% CI: 2.23, 6.92), respectively.

#### Eggs

**Fig 13:**
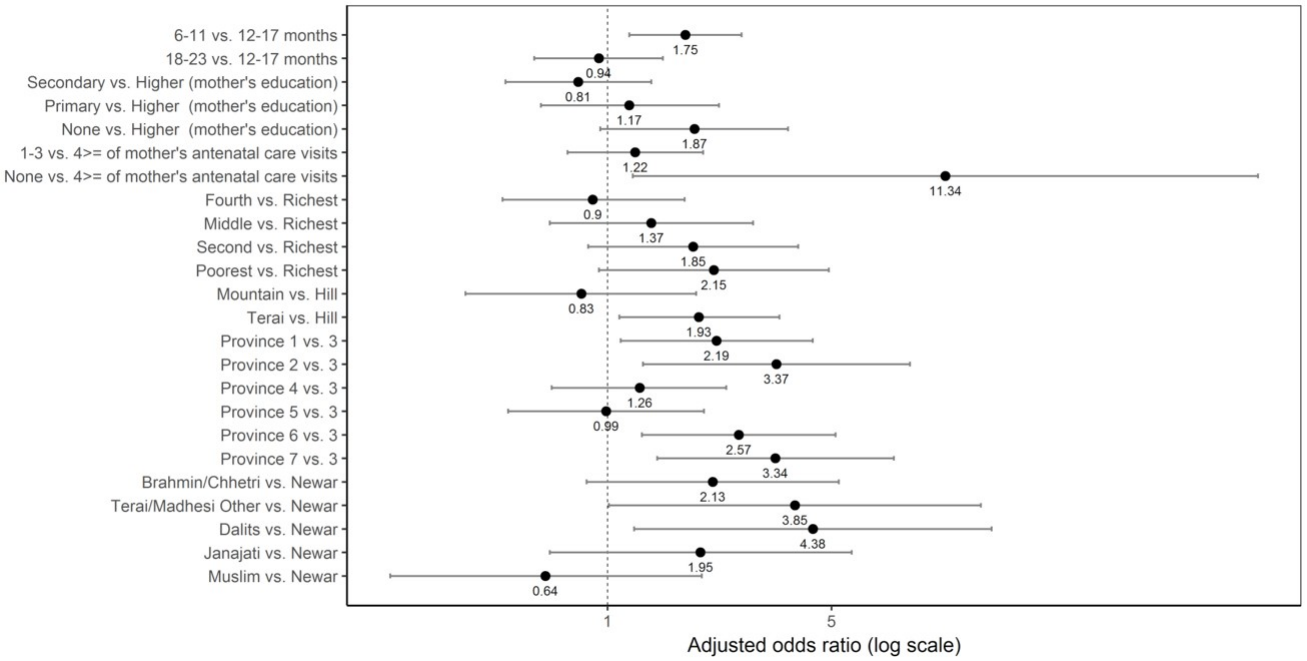
Odds ratio plot with 95% confidence intervals for not consuming eggs among Nepalese children aged 6 to 23 months.

Sociodemographic characteristics significantly associated with not consuming eggs were age, mother’s antenatal care visits, ecological region, province, and caste/ethnic groups after adjusting for the other variables (Fig 13). The odds of not consuming eggs was higher among children aged 6–11 months as compared to those aged 12–17 months, with an adjusted odds ratio of 1.75 (95% CI 1.17, 2.62). Children from Province 1 (adjusted OR = 2.19; 95% CI 1.10, 4.37), Province 2 (adjusted OR = 3.37; 95% CI 1.29, 8.79), Province 6 (adjusted OR = 2.57; 95% CI 1.28, 5.15), and Province 7 (adjusted OR = 3.34; 95% CI 1.43, 7.83) had higher odds of not consuming eggs compared to those from Province 3. Compared to children from Newar, those from Terai/Madhesi Other and Dalits had higher odds of not consuming eggs, with an adjusted odds ratio of 3.85 (95% CI: 1.01, 14.64) and 4.38 (95% CI: 1.21, 15.80), respectively.

#### Vitamin-A rich fruits and vegetables

**Fig 14:**
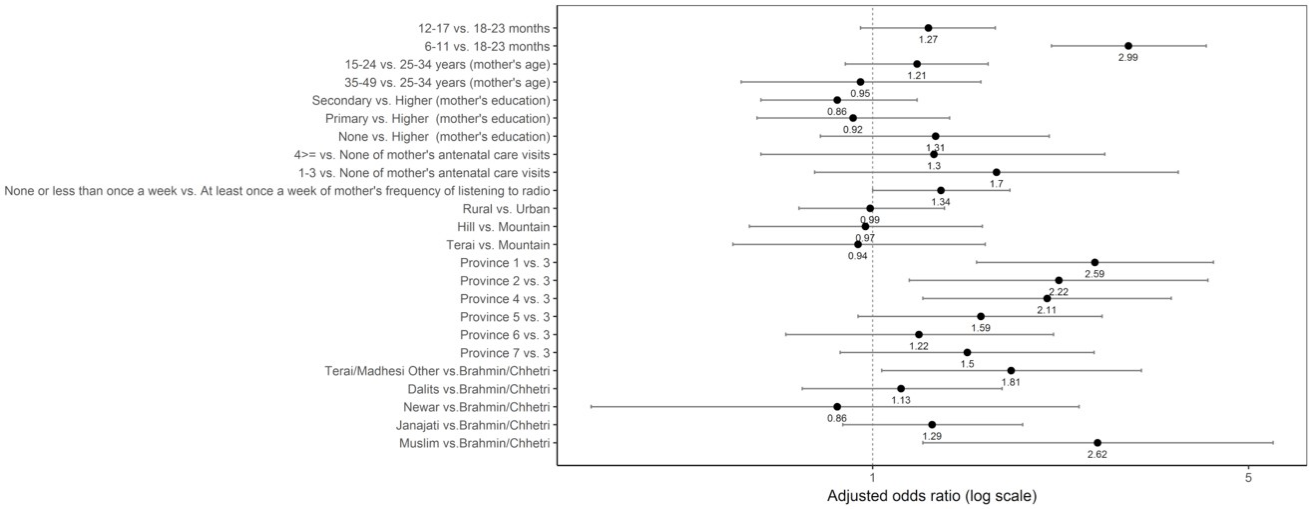
Odds ratio plot with 95% confidence intervals for not consuming vitamin-A rich fruits and vegetables among Nepalese children aged 6 to 23 months.

Age, province, and caste/ethnic groups were significantly associated with not consuming vitamin-A rich fruits and vegetables (Fig 14). The odds of not consuming vitamin-A rich fruits and vegetables was higher among children aged 6–11 months compared to those aged 18–23 months (adjusted OR = 2.99; 95% CI 2.15, 4.17). Compared to children from Province 3, those from Province 1 (adjusted OR = 2.59; 95% CI 1.56, 4.30), Province 2 (adjusted OR = 2.22; 95% CI 1.17, 4.20), and Province 4 (adjusted OR = 2.11; 95% CI 1.24, 3.59) had higher odds of not consuming vitamin-A rich fruits and vegetables. The odds of not consuming vitamin-A rich fruits and vegetables was higher among children from with Terai/Madhesi Other and Muslim, with an adjusted odds ratio of 1.81 (95% CI: 1.04, 3.16) and 2.62 (95% CI: 1.24, 5.55), respectively, compared to those from Brahmin/Chhetri.

#### Other fruits and vegetables

**Fig 15:**
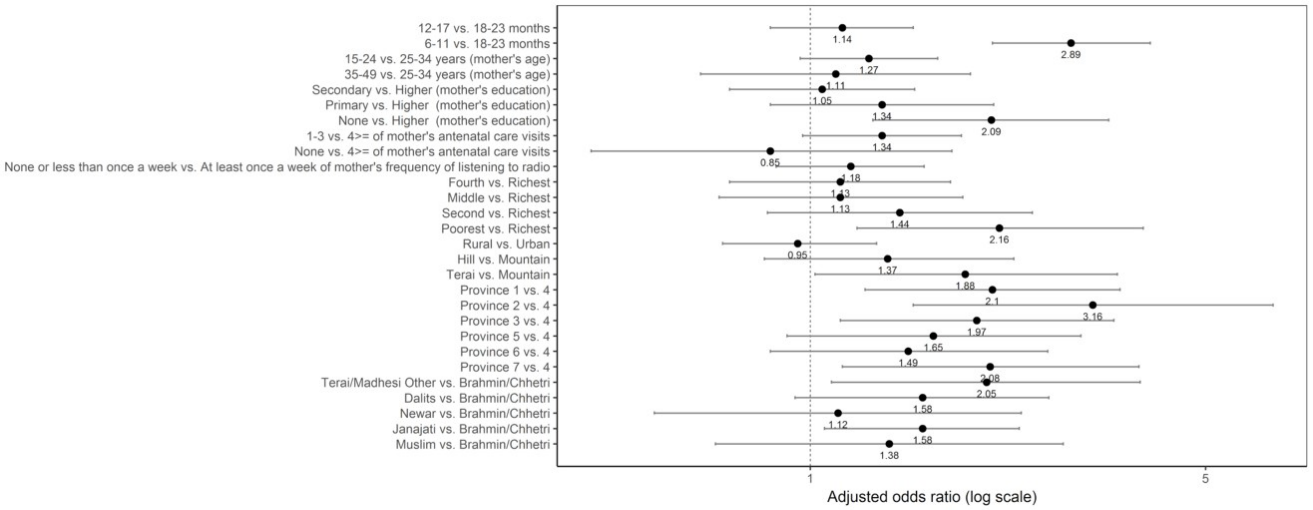
Odds ratio plot with 95% confidence intervals for not consuming other fruits and vegetables among Nepalese children aged 6 to 23 months.

Sociodemographic characteristics significantly associated with not consuming other fruits and vegetables were age, mother’s education, the wealth quintile, ecological region, province, and caste/ethnic groups (Fig 15). Children 6–11 months had higher odds of not consuming other fruits and vegetables compared to those 18–23 months (adjusted OR = 2.89; 95% CI 2.10, 3.99). Compared to children in the richest quintile, those in the poorest quintile had higher odds of not consuming other fruits and vegetables (adjusted OR = 2.16; 95% CI 1.21, 3.88). Children from Province 1 (adjusted OR = 2.10; 95% CI 1.25, 3.53), Province 2 (adjusted OR = 3.16; 95% CI 1.52, 6.58), Province 3 (adjusted OR = 1.97; 95% CI 1.13, 3.44), and Province 7 (adjusted OR = 2.08; 95% CI 1.14, 3.81) had higher odds of not consuming other fruits and vegetables compared to those from Province 4. The odds of not consuming other fruits and vegetables were higher among children from Terai/Madhesi Other (adjusted OR = 2.05; 95% CI 1.09, 3.83) and Janajati (adjusted OR = 1.58; 95% CI 1.06, 2.34) compared to those from Brahmin/Chhetri.

#### Minimum dietary diversity

**Fig 16:**
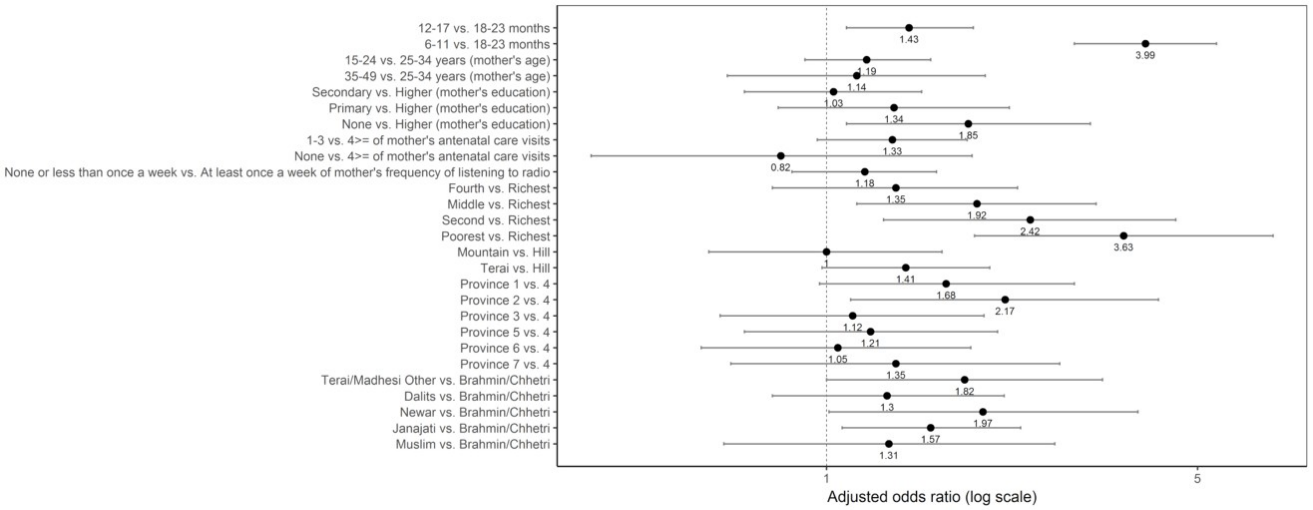
Odds ratio plot with 95% confidence intervals for not meeting the minimum dietary diversity among Nepalese children aged 6 to 23 months.

Age, mother’s education, the wealth quintile, province, and caste/ethnic groups were associated with not meeting the MDD (Fig 16). Compared to children aged 18–23 months, those aged 6–11 months (adjusted OR = 3.99; 95% CI 2.93, 5.43) and 12–17 months (adjusted OR = 1.43; 95% CI 1.09–1.89) had higher odds of not meeting the MDD. Children in the middle quintile (adjusted OR = 1.92; 95% CI 1.14, 3.22), second quintile (adjusted OR = 2.42; 95% CI 1.28, 4.55), and poorest quintile (adjusted OR = 3.63; 95% CI 1.90, 6.94) had higher odds of not meeting the MDD compared to those in the richest quintile. The odds of not meeting the MDD was significantly higher among children from Province 2 compared to those from Province 4 (adjusted OR = 2.17; 95% CI 1.11–4.22). Compared to children from Brahmin/Chhetri, those from Newar and Janajati had higher odds of not meeting the MDD, with an adjusted odds ratio of 1.97 (95% CI: 1.01, 3.86) and 1.57 (95% CI: 1.07, 2.32), respectively.

## Discussion

Food and feeding practices from birth to age two have a profound impact on the rest of a child’s life [3]. Our study examined the consumption rate of seven food groups and the MDD distribution among infants and young children aged 6–23 months by individual, household, and community level factors in Nepal. We also identified the factors associated with inadequate food group consumption and the MDD. The consumption rate of the food groups ranged from 13.6% for eggs to 92.1% for grains, roots and tubers and less than half (46.5%) of children met the MDD. Age and caste/ethnic groups were consistently associated with not consuming foods from the seven food groups and not meeting the MDD. Wealth was associated with not consuming legumes and nuts, dairy products, flesh foods, and other fruits and vegetables and not meeting the MDD. Province was associated with not consuming grains, roots and tubers, legumes and nuts, eggs, vitamin-A rich fruits and vegetables, and other fruits and vegetables and not meeting the MDD.

The least consumed food group was eggs followed by flesh foods and the percentage of consumption were 13.6% and 25.1%, respectively. Data showed that the consumption of flesh foods and eggs among children in Nepal has improved over the last several years. According to a study using the 2006 Nepal DHS, the percentages of consumption of eggs and flesh foods among young children was 6.4% and 17.3%, respectively [17]. Another study using the 2011 Nepal DHS showed that the consumption rates of eggs and flesh foods was 9.6% and 18.1%, respectively [18]. Despite the improvement, the consumption rate of these food groups is still low. Compared to other countries in South Asia, the percentage of food consumption among children in Bangladesh was higher, as nearly half of young children received eggs, and fish or meat [7]. In Sri Lanka, 22% of children consumed eggs and 62% of children consumed flesh foods [9]. It was reported that mothers in Nepal considered that cereal-based foods were nutritious for their babies and failed to feed their babies meat, fish, or eggs. [26]. Similarly, another report showed that a high proportion of households did not consume any meat, fish, eggs or fruit in Nepal [27]. Nutrition programs to encourage these least consumed food groups would improve overall dietary diversity of young children in Nepal. Cash transfer programs may be considered as some evidences showed that these may help increase spending on animal source foods and have a positive effect on the resources for food security [28, 29].

Age was significantly associated with not consuming foods from the seven food groups and not meeting the MDD. Children 6–11 months of age had higher odds of not receiving seven food groups and the MDD than older children. These findings are consistent with previous studies in Nepal [20] and other countries [7, 8, 9, 10, 11, 12, 13]. Globally, less than a quarter of children 6 to 11 months of age received four or more food groups a day, whereas nearly half of children 18 to 23 months of age received them [3]. This may be due to mothers’ perceptions toward IYCF. Research examining food practices and beliefs in Nepal found that many mothers thought children before the age of 1 year should avoid animal-source foods [30] and mothers gave pulses more frequently to children above 24 months old than those less than 8 months [26]. National strategies and programs targeting mothers with younger children to promote optimal food and feeding practices are necessary.

Caste/ethnicity was consistently associated with not consuming foods from the seven food groups and not meeting the MDD. In our study, children from Terai/Madhesi Other had higher odds of not receiving seven food groups. Children from Dalits had higher odds of not consuming grains, roots and tubers, dairy products, and eggs and not meeting the MDD. Children from Janajati had higher odds of not consuming grains, roots and tubers, legumes and nuts, dairy products, and other fruits and vegetables and not meeting the MDD. Similar findings were reported in a previous study in Nepal which showed that children from Dalits and Janajati had higher odds ratio of not meeting the MDD than those from Brahmin/Chhetri [20]. Reports on health and caste/ethnicity in Nepal showed that Dalits, Muslims, and Terai/Madhesi Other castes, constituting 28% of Nepal’s population, had poor health and nutrition indicators [22, 23]. In our study, children from Brahmin/Chhetri were more likely to receive different food groups than those from other caste/ethnic groups, except for flesh foods. This may reflect their religious beliefs [26]. According to a study on cultural and environmental factors regarding IYCF, mothers from Brahmin/Chhetri perceived poultry to be unclean [30]. Overall, low consumption among children from certain caste/ethnic groups may indicate inequality in diverse diets across ethnicity and caste. Nutritional programs need to consider various cultural practices and conditions of caste/ethnic groups.

In this study, children from Province 1 had higher odds of not consuming legumes and nuts, eggs, vitamin-A rich fruits and vegetables, and other fruits and vegetables. Children from Province 2 had higher odds of not consuming eggs, vitamin-A rich fruits and vegetables, and other fruits and vegetables, and not meeting the MDD. The 2016 Nepal DHS report showed that nutritional status was poor among children from Provinces 1 and 2 [21]. Province 2 (14.4%) and Province 1 (11.8%) had a higher proportion of wasted children under the age of 5 years than other provinces [21]. The proportion of children receiving the minimum acceptable diet was the lowest in Province 2 (20.4%), followed by Province 1 (33.9%) [21]. In addition, the prevalence of anemia was the highest among children aged 6–59 months from Province 2 (59.4%), followed by Province 1 (55.2%) [21]. Meanwhile, regional variance was reported as one of the determinant factors of IYCF practices in previous studies [7, 8, 11, 12, 18]. It may be attributable to diverse food cultures or beliefs about foods across the regions [8]. Further studies to identify determinant factors of food consumption in different provinces would be helpful to tailor policies and educational material to encourage diverse diets.

Children from the poorest quintile had higher odds of not consuming legumes and nuts, dairy products, flesh foods, and other fruits and vegetables, and not meeting the MDD. It may be possible that family members from poor households mainly consume cereals and do not have diverse diets [31]. A lack of financial resources may be a barrier to accessing nutritious foods, thus, mothers from low-income households are less likely to give their children diverse diets compared to those from high-income households [12, 17]. Our study supports the previous studies indicating the positive impact of wealth on diverse diets [7, 8, 9, 10, 11, 12, 13, 15,17].

Several studies reported the positive impact of maternal education [7, 8, 9, 13, 15, 17, 20, 32] and antenatal care visits [9, 17, 20] on healthy IYCF practices. However, the positive association between the two factors and food group consumption was not consistently found in this study. The positive association regarding mother’s education was found only in consumption of dairy products and other fruits and vegetables. Similarly, the positive association was only identified between mother’s antenatal care visits and egg consumption. Mothers with more antenatal care visits may have better access to health and nutrition information since IYCF counselling and pertinent messages are delivered during the visits [20, 33]. It may also be possible that educated mothers have more information and understand educational messages well [15]. Though our study did not find a strong association, further research would be necessary to examine the associations between these factors and food group consumption among young children in Nepal.

The dietary diversity indicator is a useful tool to identify populations at risk and design interventions. However, disaggregated data by food groups should not be neglected. It is important to assess consumption of each food group when designing nutrition interventions. Similarly, a study on a dietary diversity indicator recognized its simplicity for measurement purpose yet recommended a food-based approach for education purposes [34]. Meeting the MDD does not mean that children have an adequate diet. A study in Pakistan found that legumes and nuts, vitamin A-rich fruits and vegetables, and flesh foods were not consumed among 45–85% of the children who received the MDD [10]. Developing tools to measure food group consumption among children may be helpful for designing nutrition intervention in order to promote a diversified diet.

The present study has the following limitations and strengths. First, as the data source of this study is a cross-sectional survey, associations were estimation rather than causality. Second, the amount and the quality of food given to children were not considered, only that they had consumed it. Third, recall bias should be considered as the data were obtained based on maternal recall. Lastly, though the 2016 Nepal DHS was conducted a year after a 7.8 magnitude earthquake, the impact of the earthquake might influence the survey response [21]. Nevertheless, the strengths of this study include that it used the latest national survey and thus the results could be generalized at the national level. Furthermore, the study assessed the association between sociodemographic factors and inadequate food group consumption and dietary diversity among children, which provided a better understanding of intake practices. National policies and programs along with further studies could be designed based on the results of this study.

In conclusion, consumption of diverse food groups and dietary diversity among children aged 6–23 months in Nepal need improvement and its consumption rate varied with sociodemographic factors indicating inequities in dietary diversity. The younger age groups and children from Terai/Madhesi Other and Janajati had higher odds of not consuming diverse food groups and not meeting the MDD. The findings suggest that national policies and programs should be formulated with an equity lens to promote a diversified diet by considering different sociodemographic factors.
